# Association of breast cancer with human papillomavirus (HPV) infection in Northeast Brazil: molecular evidence

**DOI:** 10.6061/clinics/2018/e465

**Published:** 2018-10-05

**Authors:** José Roosevelt Cavalcante, Luiz Gonzaga Porto Pinheiro, Paulo Roberto Carvalho de Almeida, Márcia Valéria Pitombeira Ferreira, Gizele Almada Cruz, Thales Alves Campelo, Caroliny Soares Silva, Luana Nepomuceno Gondim Costa Lima, Bruno Masato Kitagawa de Oliveira, Lara Mulato Lima, Laura Magda Costa Feitosa, Agostinho Câmara Pinheiro, Cristiane Cunha Frota

**Affiliations:** I Departamento de Cirurgia e Maternidade Escola Assis Chateaubriand, Faculdade de Medicina, Universidade Federal Ceara (UFC), Fortaleza, CE, BR; II Departamento de Patologia e Medicina Legal, Faculdade de Medicina, Universidade Federal Ceara (UFC), Fortaleza, CE, BR; III Instituto Evandro Chagas, Belem, PA, BR; IV Faculdade de Medicina, Universidade Federal Ceara (UFC), Fortaleza, CE, BR

**Keywords:** Breast Neoplasms, Human Papillomavirus, HPV, Molecular Typing, Breast Cancer

## Abstract

**OBJECTIVE:**

The aim of this study is to investigate the presence of human papillomavirus DNA and genotypes in breast cancer and normal breast tissue samples obtained from women from the northeast region of Brazil.

**METHOD:**

One hundred three breast cancer samples and 95 normal breast samples, as the non-malignant controls, were studied. DNA extraction was verified by human beta-globin gene amplification, and polymerase chain reaction was conducted based on HPV L1-specific consensus primers MY09/MY11 and GP5+/GP6+, followed by nested multiplex polymerase chain reaction with type-specific primers for the E6/E7 consensus region.

**RESULTS:**

Human papillomavirus DNA was detected in 51 (49.5%) breast carcinoma samples and 15 (15.8%) normal breast samples (*p*<0.0001). Human papillomavirus genotypes 6 and 11 were identified in 15.2% of all samples.

**CONCLUSIONS:**

The high frequency of human papillomavirus infection in breast cancer samples indicates a potential role of this virus in breast carcinogenesis in the studied participants.

## INTRODUCTION

Breast cancer (BC) is the most common tumour among women throughout the world in both developed and developing countries. Approximately 1.79 million new cases of BC were predicted for 2015, with an estimated death count of 560,407 for the same year 1. In Brazil, approximately 57,960 new cases of BC were expected in 2016, which is equivalent to an estimated risk of 5,620 for each 100,000 women 2.

Several risk factors are described for BC, such as age, family history, weight, physical activity, oral contraceptive use, and alcohol consumption 3. Some viruses may have a role in BC, including mouse mammary tumour virus (MMTV), bovine leukaemia virus (BLV), human papilloma viruses (HPVs), and Epstein-Barr virus (EBV) 4. Regarding HPV and BC, it has been demonstrated that HPV types 16 and 18 can immortalize normal breast (NB) epithelium 5. Reinforcing the putative carcinogenesis in BC, HPV has several ways of subverting an effective immune response that may contribute to persistence 6.

HPV belongs to the *Papillomaviridae* family, which is classified into five genera: α-papillomavirus, β-papillomavirus, γ-papillomavirus, µ-papillomavirus, and ν-papillomavirus 7,8. HPVs have small, circular, double-stranded DNA genomes that contain three oncogenes, E5, E6, and E7; two regulatory proteins, E1 and E2; and two structural proteins, L1 and L2 7,8. The HPV E6 and E7 proteins are known for blocking *p*Rb and *p*53 tumour suppressor genes, contributing to oncogenesis 6. The conserved region for the capsid protein L1 ORF is used to classify HPV types to different genera or virus species within a genus 7,8. Polymerase chain reaction (PCR)-based assays using highly conserved amino acid residues in the L1 ORF (MY09 and MY11 primers) segment can be used to identify a large number of HPV types 9,10. Novel HPV types are confirmed and deposited in The International Human Papillomavirus (HPV) Reference Center 7,8,10.

HPV is present in more than 99% of cervical cancer cases and is considered one of the main risk factors for many penile, vulvar, vaginal, and anal carcinomas and a growing fraction of head and neck squamous cell carcinomas 3,6,9,11,12. HPV DNA sequences were first reported in BC samples in 1992 13. Subsequently, several studies have documented the presence of HPV DNA in BC tissues 12,14,15. Despite the emergence of HPV in BC as a major public health issue worldwide, only one previous study has been reported in Brazil; it was conducted in Porto Alegre and found HPV 16 and 18 in 24.5% of the cases 16. However, there are geographical differences in the distribution of HPV types found in BC among populations 14,17,18. Therefore, this study aimed to identify HPV DNA and genotypes in BC and NB samples obtained from women from the Northeast Region of Brazil.

## MATERIALS AND METHODS

### Study population

This was a cross-sectional study conducted from July 2014 to December 2015 in Fortaleza, Northeast Brazil. BC biopsy samples from women were collected from histopathological files from the Department of Pathology and Legal Medicine, Federal University of Ceará, Fortaleza, Brazil. NB tissues were collected at the Biopse Pathology Laboratory, Fortaleza, Brazil. A total of 198 specimens were selected, of which 103 were primary BC samples and 95 were NB tissues with no malignancy. The controls were breast samples obtained from cosmetic reduction mammoplasties of women without a prior history of breast neoplasm.

For the distribution of results, the age groups defined in this study were based on parameters recognized and adopted by mastologists regarding the initiation of breast screening in women (40 years) 1 and a study on the mean age of menopause in Brazil (51 years) according to Pedro et al. 19. Thus, in this study, the women were stratified into three age groups: younger than 42 years, between 42 and 51 years, and older than 51 years.

This project was approved by the Ethical Committee of Maternity School Assis Chateaubriand (MEAC), Federal University of Ceará (protocol 728,048/2014), and the guidelines of the ethical committee were followed in conducting this study. Legal transfer of the custodianship of the tissues samples was obtained from the Department of Pathology and Forensic Medicine (DPML) of the Federal University of Ceará, Fortaleza, Brazil.

### Inclusion and exclusion criteria

Patients were eligible for study participation if they met all of the following criteria: new diagnosis of BC confirmed by histopathology for the BC group or no malignancy with no history of BC for the NB group and positivity of the sample to the human beta-globin (β-globin) gene. Patients who received neoadjuvant chemotherapy or radiation therapy were excluded from the study.

### Tissue samples

A skin punch was used to collect a 3-mm-diameter core from formalin-fixed paraffin-embedded tissue samples. All tissue sections were taken after removal of the surgical specimen. Prior to this, haematoxylin and eosin (HE) staining was performed to identify the tumour site in each tissue block. In the NB control samples, areas containing ducts and mammary lobules were selected. The tissue fragments obtained from BC cases and NB controls were placed in 1.5-ml labelled tubes and stored at -20°C until processing.

### Deparaffinization of tissue sections

Before deparaffinization, excess paraffin was removed from each tissue section using sterile blades. Deparaffinization of embedded samples was conducted following the protocol described in Nascimento et al. 20. At the end of the process, the tissues were rehydrated in 1 ml of ultrapure water.

### Genomic DNA extraction

DNA was extracted using the PureLink^TM^ Genomic DNA Mini Kit (Life Technologies Corporation, Carlsbad, USA) following the manufacturer's guidelines. Purity of the extracted DNA was estimated from the ratio between spectrophotometric absorptions at 260 and 280 nm (OD_260_/OD_280_).

All samples were examined for DNA integrity via PCR amplification of the human β-globin housekeeping gene. DNA from the breast tissue samples from all participants were probed for a 268-bp product of the β-globin gene via PCR following the protocol described in Saiki et al. 21.

### PCR amplification

All β-globin-positive isolates were screened for the HPV L1 conserved region. Nested PCR was conducted with the outer consensus primers MY09/MY11 (product size 450 bp), followed by amplification with the inner primers GP5+/GP6+ to amplify a 150-bp fragment. The PCR amplification conditions were as previously described 18. Illustra PuReTaq Ready-To-Go^TM^ PCR Beads predispensed in 0.25-ml tubes (GE Healthcare, Little Chalfont, UK) were used for PCR amplification.

### HPV typing by nested multiplex PCR

GP-E6 3F/5B/6B and type-specific primer sets were used for nested PCR for the exact typing of HPV. The type-specific primers were combined in two multiplex PCR amplifications for identifying genotypes corresponding to low and high oncogenic risk. Primer mix 1 targeted types 16, 18, 31, 45, and 59, whereas primer mix 2 targeted types 33, 6/11, 52, 56, and 58. Primer mix 1 generates fragments with lengths of 457, 322, 263, 215, and 151 bp corresponding to HPV types 16, 18, 31, 59, and 45, respectively. Primer mix 2 amplifies fragments with lengths of 398, 334, 274, 274, 229, and 181 bp corresponding to HPV types 33, 6/11, 58, 52, and 56, respectively. HPV typing was conducted as described in Sotlar et al. 22.

All procedures were conducted by the same technician using the same methods. False-positive amplifications were addressed using individual sterile sectioning blades for cutting the paraffin-embedded tissues, and sterile glassware were used for all samples. Physical separation of areas for sample handling, PCR preparation, and PCR analysis was diligently practised throughout the study.

### DNA sequencing

PCR products derived with the E6/E7 consensus primers were purified using the QIAquick PCR Purification Kit (Qiagen, Hilden, Germany) prior to sequencing with an Applied Biosystems DNA Sequencer (Applied Biosystems^TM^, Carlsbad, USA) using a BigDye Terminator Cycle Sequencing kit. Sequences were analysed using SecScape software v.2.7 (Applied Biosystems^TM^, Carlsbad, USA) and BioEdit Sequence Alignment Editor version 7.2.5 (BioEdit, Carlsbad, USA). The reference sequence for HPV type 11 isolate 83A.11 (GenBank accession KU298879.1) and the HPV-16 E6/E7 fusion protein gene (GenBank accession FJ229356.1) were used to align the sequences.

### Statistical analysis

The data were entered into Microsoft® Excel 2013 and transferred to GraphPad Prism 5 software statistical software (GraphPad Software, San Diego, USA) to conduct descriptive and bivariate analyses. The chi-square test and Fisher's exact two-tailed test were performed, and differences were considered significant at values of *p*<0.05. Associations of HPV PCR positivity with BC and normal breast samples were performed using Student's t-test, estimating the crude odds ratios (ORs) with their respective 95% confidence intervals (CIs).

## RESULTS

The age ranges were from 27 to 97 years (mean of 55.2±13.50 years) for the BC patients and from 24 to 84 years (mean of 41.4±11.67 years) for the NB participants. Amplification of a 268-bp fragment of the human β-globin gene was observed in all studied samples, confirming that all tissue samples had amplifiable DNA in the absence of any inhibition. The distribution of HPV-positive samples based on the different PCR primer sets is shown in Table 1. In the MY/GP PCR assay, 43.7% (45/103) of the BC samples were positive for HPV DNA, whereas 7.4% (7/95) of the NB samples were positive for HPV DNA (*p*<0.0001, OR=9.75, 95% CI 4.12-23.11). In the E6/E7 assay, 10.7% (11/103) of the BC samples were positive for HPV DNA, among which, five samples had already been identified as positive with the MY/GP primers. Moreover, 8.4% (8/95) of the NB samples were positive for HPV DNA in the E6/E7 assay. Notably, none of these NB samples were positive with the MY/GP primers. Thus, the overall positivities for HPV DNA were 49.5% (51/103) and 15.8% (15/95) in the BC and NB samples, respectively (*p*<0.0001, OR=5.23, 95% CI 2.67-10.26).

The results for HPV DNA positivity for both the BC and NB samples were stratified by age groups (Table 2). HPV DNA was identified in 31% (22/71) of participants who were younger than 42 years – 77.8% (14/18) of the BC group and 15% (8/53) of the NB group (*p*<0.0001). In the participants aged 42-51 years, HPV DNA was identified in 33.3% (16/48) of all participants – 52.2% (12/23) of the BC group and 16% (4/25) of the NB group (*p*=0.0135). In the participants older than 51 years, HPV DNA was identified in 35.4% (28/79) of all participants – 40.3% (25/62) of the BC group and 17.6% (3/17) of the NB group (*p*=0.956). In the BC group, HPV infection was more common in women who were younger than 42 years than in women who were older 51 years (*p*=0.0070). However, no differences were observed regarding HPV infection status and different age groups in the NB group.

Regarding genotype identification (Table 3), HPV 6/11 was the most common type (15.2%; 10/66) among all participants. Seven (13.7%) BC samples and three (20%) NB samples were positive for HPV type 6/11, followed by two (3.9%) BC samples and two (13.3%) NB samples that were positive for HPV type 18. Additionally, one (6.7%) NB sample and another sample positive for HPV type 52 (6.7%) were identified as positive for HPV type 31. HPV type 33 was also identified in two (3.9%) BC samples and in one (6.7%) NB sample. Non-genotyped HPV types (HPVX) were identified in 78.5% (40/51) of BC samples and in 46.6% (7/15) of NB samples. Regarding samples that were positive for HPV types corresponding to a high oncogenic risk, 7.8% (4/51) were in the BC group and 33.3% (5/15) were in the NB group. Furthermore, for samples positive for HPV types corresponding to a low oncogenic risk, 13.7% (7/51) were in the BC group and 20% (3/15) were in the NB group.

Regarding the age prevalence of the identified HPV types, HPV 6/11 (n=10) was prevalent across all ages, with a prevalence peak in the >51 years age group (five cases), followed by three patients younger than 42 years and two patients in the 42-51 years age group. HPV 18 (n=4) was prevalent in younger participants, presenting in three participants in the 42-51 years age group and one participant who was 25 years old. HPV 33 (n=3) was equally distributed in the three age groups. HPV 31 and HPV 52 were detected in the younger group (<42 years). In addition, a 64-year-old patient was positive for HPV 45 (data not shown).

**Figure 1:**
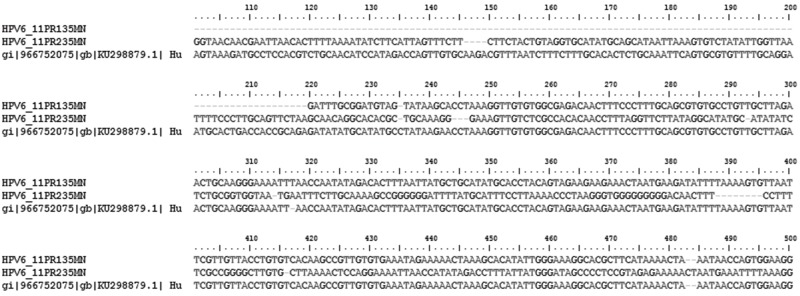
Confirmation of amplification of DNA of HPV extracted from tissue samples of normal breast participants. PCR products of the HPV type 11 isolate 83A.11 sequence (accession No. KU298879.1) were sequenced using an ABI 3130 Genetic Analyser (Applied Biosystems^TM^, Carlsbad, CA, USA) and were analysed using the BioEdit Sequence Alignment Editor Program (BioEdit, Carlsbad, CA, USA).

BLAST search results confirmed that product sequences identified in the positive control sample and in positive NB and BC samples corresponded to HPV DNA. The DNA sequences corresponding to the PCR products obtained using E6/E7 consensus primers aligned with the HPV-16 E6/E7 fusion protein gene (FJ229356.1) and the HPV type 11 isolate 83A.11 sequence (KU298879.1), with 100% homology observed for all sequences (Figure 1).

## DISCUSSION

This study identified a very high PCR positivity of HPV DNA in BC tissue samples. HPV DNA was detected in 49.5% of BC cases and in 15.8% of NB controls, indicating a statistically significant difference between these two groups. Our results support a potential relationship between HPV infection and breast carcinoma. The OR for HPV positivity was 5.2-fold higher in BC cases than in NB cases. Our results are similar to the findings of a study conducted in Alicante, Spain, which also used embedded BC tissues 14; however, our study identified lower proportions of HPV positivity than did the Spanish study (51.8% and 26.3% for the cases and controls, respectively). In contrast, the values identified in our study were much higher than those described in a meta-analysis of 29 published studies 23, in which the prevalence rates of HPV infection were 23% in BC cases and 12.9% in NB cases. This is the first study conducted in the Northeast Region of Brazil that investigated the association between HPV positivity and BC. A few studies had previously investigated the association between HPV infection and BC in South America, one of them being in Brazil 16 and the other in Argentina 24.

In this study, we used two consensus primer sets and 11 type-specific primers and identified HPV 6/11 as the most frequent (15.2%) viral genotype in the studied population. In Brazil, the HPV genotypes used for vaccination are 6, 11, 16, and 18. However, in the state of Ceará, immunization against HPV was only started in 2014 for girls between 9 and 14 years of age and in 2017 for women between 9 and 45 years and men between 9 and 26 years of age. HPV types 6 and 11 are classified as having a low oncogenic potential for the lower genital tract. In addition, these viral genotypes are generally associated with benign genital warts, known as condylomas 3,7. Similarly, a study conducted in Heidelberg, Germany, identified HPV types 6 and 11 as the most frequent genotypes in their study of mammary lesions and invasive breast carcinomas 3. In a similar way, a study conducted in Rio de Janeiro with cervical cancer patients 25 have found low-risk types in tumour samples. Another study also indicated that women who develop viral warts are at a significantly higher risk of developing BC 26; low-risk HPV types are causal agents of genital warts 3,7. In addition, a study conducted with oral cancer in 187 patients had also suggested that separating high- and low-risk groups based on the HPV status of other body parts might not be appropriate 27. Nevertheless, the majority of the studies have reported the presence of high-risk HPV in BC samples 11,13,16,17,28.

Notably, we did not detect HPV type 16, which was shown to be prevalent in previous studies on BCs 29-31, in either BC or NB samples. In the southern region of Brazil, a study conducted with paraffin-embedded breast carcinoma samples using specific primers for two HPV types, namely, types 16 and 18, found a predominance of HPV type 16 16. However, two different studies conducted in Australia on BC samples, one in Sydney 30 and the other in Queensland 11, found HPV type 18 in 48% and 50% of samples, respectively, but neither identified HPV type 16. Therefore, there is a broad range of distribution of HPV genotypes in BC samples, depending on the geographical region. Similarly, studies conducted in China 18 and Syria 28 identified HPV 33 as the most frequent genotype in BC samples, while a study conducted in Canada 32 identified HPV 16 as the predominant genotype. Lawson et al. 33 had already demonstrated that viral genotypes can vary based on different geographic locations and that this variation may result in differential oncogenicity.

Considering the size of a country such as Brazil, it is possible to explain the diversity based on the geographic distribution of this virus. However, the only previous study conducted in Brazil 16 used two primer sets specific for types 16 and 18, which may have made the detection of other genotypes difficult. The introduction of new molecular detection technologies has enabled the discovery of new HPV types, and it is now estimated that there are at least 400 different HPV types 10. Thus, assays using type-specific primers have limitations that must be considered, as these assays may not reveal the presence of all genotypes in samples.

The distribution of HPV DNA in the three age groups, from the younger (<42 years) group to the older (>51 years) group, demonstrated a decrease in the proportion of HPV-positive cases with increasing age (77.8%, 52.2%, and 40.3% corresponding to age groups <42 years, 42-51 years, and >51 years, respectively), with a statistically significant difference between the group younger than 42 years and the group older than 51 years (*p*=0.0070). Thus, the likelihood HPV infection decreases with age. A study conducted in Lublin, Poland 15, with a similar design as ours, demonstrated a direct correlation between the presence of HPV DNA and patient age. In fact, the observation of higher frequency of HPV positivity in younger women has been supported by several studies, which demonstrated an association between HPV infection in younger BC patients and the frequency of their sexual activity 28,33,34.

Several mechanisms of viral transmission have been proposed for BC, including skin-to-skin contact and sexual activity 7,14,17,35. In addition, the higher frequency of HPV infection in BC patients younger than 42 years may be explained by the modulation of carcinogenesis by female hormones. Transgenic mice that are infected with HPV in the presence of oestrogen treatment have been shown to have decreased oestrogen receptor-alpha (ERα) expression 36. Thus, it has been hypothesized that HPV drives mechanisms to promote cell proliferation 36. In addition, several genes have been identified as being oestrogen-responsive in BC studies following a decrease in ERα expression and an increase in ERβ expression 37.

Several risk factors, including young age at onset of sexual activity and high number of sexual partners, are related to exposure to HPV. Furthermore, oral contraceptive use is associated with progression from HPV infection to cancer. For example, the use of contraceptives may contribute to increased sexual behaviour in women and can increase their exposure to and risk of HPV infections 38. The presence of HPV DNA in NB samples might be explained by the notion that HPV infection precedes cancer diagnosis, as already mentioned by other authors 14,17,31,35.

In the current study, HPV DNA was detected in biopsies of archived embedded paraffin tissues. However, all these tissue samples were previously fixed with 10% formaldehyde, which can fragment DNA, modify nucleotide bases and generate internal cross-linking between DNA and proteins 39. According to Karlsen et al. 40, eight hours of fixation is sufficient to inhibit the amplification of DNA products over 421 bp in size. Moreover, the same authors demonstrated that fixation for longer than one week can influence the amplification of DNA fragments over 200 bp in size. Furthermore, Baay et al. 41 demonstrated a correlation between the efficiency of the PCR assay and the length of the PCR product in paraffin-embedded tissue samples.

In summary, we have demonstrated the presence of HPV DNA in a high proportion of BC and NB tissues from women from Northeast Brazil. In addition, we also demonstrated a high frequency of the low-risk viral genotypes, namely, HPV types 6 and 11, in these BC and NB samples. Our results demonstrated the presence of HPV in a significant proportion of malignant breast and non-malignant tissues. The presence of low and high-risk types suggests a potential role of this virus in breast carcinogenesis in the studied participants. Thus, further studies are needed to understand the role of HPV infection in breast carcinogenesis, including among women vaccinated against high-risk HPV types.

## AUTHOR CONTRIBUTIONS

Cavalcante JR designed the study, gathered and analyzed the data, and wrote the manuscript. Pinheiro LG designed the study and analyzed and interpreted the data. Almeida PR and Ferreira MV performed all histopathological analyses. Cruz GA, Campelo TA, Silva CS, and Lima NG conducted all molecular biological assays. Oliveira BM, Lima LM, Feitosa LM and Pinheiro AC were responsible for data acquisition. Frota CC designed the study, analyzed and interpreted the data, and wrote and critically revised the manuscript. All authors read and approved the final version of the manuscript.

## Figures and Tables

**Table 1:** HPV positivity of breast cancers and normal breasts according to consensus primer sets MY/GP and E6-E7.

Primers	Breast cancer *n*=103 (%)	Normal breast *n*=95 (%)	*p*-value^§^	OR^¶^ **(95% CI^∞^)**
MY/GP				
Positive	45 (43.7)	7 (7.4)	<0.0001	9.75 (4.12-23.11)
Negative	58 (56.3)	88 (92.6)		1
E6-E7				
Positive	11^†^ (10.7)	08 (8.4)		
Negative	92 (89.3)	87 (91.6)		
MY/GP and E6-E7^‡^				
Positive	51 (49.5)	15 (15.8)	<0.0001	5.23 (2.67-10.26)
Negative	52 (50.5)	80 (84.2)		1

^†^Five of these had already been detected with MY/GP primers; ^‡^MY/GP PCR positive, they were all added to sum up the result of 15 HPV DNA positive cases; ^§^Fisher’s test; ^¶^ OD, Odds ratio; ^∞^CI, Confidence interval.

**Table 2:** HPV positivity obtained with consensus primer sets MY/GP and E6-E7 in breast cancers and in normal breasts according to age range.

Age range (years)	Breast cancer			Normal breast		
	HPV +*n*=51 (%)	HPV - *n*=52 (%)	Total *n*=103 (%)	HPV + *n*=15 (%)	HPV - *n*=80 (%)	Total *n*= 95 (%)
<42	14 (77.8)^†^	4 (22.2)	18 (100)	8 (15)	45 (85)	53 (100)
42-51	12 (52.2)	11 (47.8)	23 (100)	4 (16)	21 (84)	25 (100)
>51	25 (40.3)^†^	37 (59.7)	62 (100)	3 (17.6)	14 (82.4)	17 (100)

^†^Comparison between the <42 years and >51 years groups, *p*=0.0070 (Fisher’s test)

**Table 3:** HPV positivity in breast cancers and normal breasts by genotypes and risk group for carcinogenesis.

HPV type	Total *n*=66 (%)	Breast cancer *n*=51 (%)	Normal breast *n*=15 (%)
Genotype			
6/11	10 (15.2)	7 (13.7)	3 (20)
18	4 (6.1)	2 (3.9)	2 (13.3)
31	1 (1.5)	-	1 (6.7)
33	3 (4.5)	2 (3.9)	1 (6.7)
31 and 52	1 (1.5)		1 (6.7)
HPVX^†^	47 (71.2)	40 (78.5)	7 (46.6)
Risk group			
High	9 (13.6)	4 (7.8)	5 (33.3)
Low	10 (15.2)	7 (13.7)	3 (20)
HPVX^†^	47 (71.2)	40 (78.5)	7 (46.7)

^†^HPVX: types not specifically detected by the primers used in this study.
